# Ultrasound neuromodulation in brain disorders: from mechanotransduction mechanisms to clinical translation

**DOI:** 10.3389/fnins.2026.1942293

**Published:** 2026-09-21

**Authors:** Rufei Zhang, Li Li, Jinzhong Tang, Si Hu, Qin Zhang, Jie Tan

**Affiliations:** 1 Acupuncture and Tuina Rehabilitation Center, The First Hospital of Hunan University of Chinese Medicine, Changsha, Hunan, China; 2 College of Acupuncture-Moxibustion, Tuina and Rehabilitation, Hunan University of Chinese Medicine, Changsha, China; 3 Medical School, Hunan University of Chinese Medicine, Changsha, Hunan, China; 4 Department of Biomedical Engineering, The Hong Kong Polytechnic University, Hong Kong, Hong Kong SAR, China

**Keywords:** brain disorders, mechanosensitive ion channels, mechanotransduction, neuroplasticity, ultrasound neuromodulation

## Abstract

Brain disorders impose a major global health burden, yet current pharmacological and neuromodulation therapies are often constrained by invasiveness, limited spatial precision, poor access to deep brain structures, or insufficient long-term efficacy. Non-invasive ultrasound neuromodulation has emerged as a promising approach combining high spatial resolution, deep tissue penetration, and reversible modulation. Its biological effects arise from multiscale mechanotransduction involving membrane deformation, mechanosensitive ion channels, intracellular calcium signaling, glial regulation, and neurovascular coupling. Together, these processes modulate neuronal excitability, synaptic plasticity, network connectivity, neuroinflammation, and cerebral perfusion. Preclinical studies support its therapeutic potential across neurological and psychiatric disorders, while early clinical studies indicate short-term tolerability and preliminary target engagement in Alzheimer’s disease, Parkinson’s disease, and depression. Emerging approaches, including individualized treatment planning, closed-loop stimulation, and sonogenetics, may further improve the precision and cellular specificity of ultrasound neuromodulation. This review summarizes the physical principles and cellular mechanisms of ultrasound neuromodulation, evaluates its therapeutic and clinical applications, and discusses key challenges and future directions, including personalized stimulation, closed-loop systems, sonogenetics, and disease-modifying strategies.

## Graphical abstract

**Figure:**
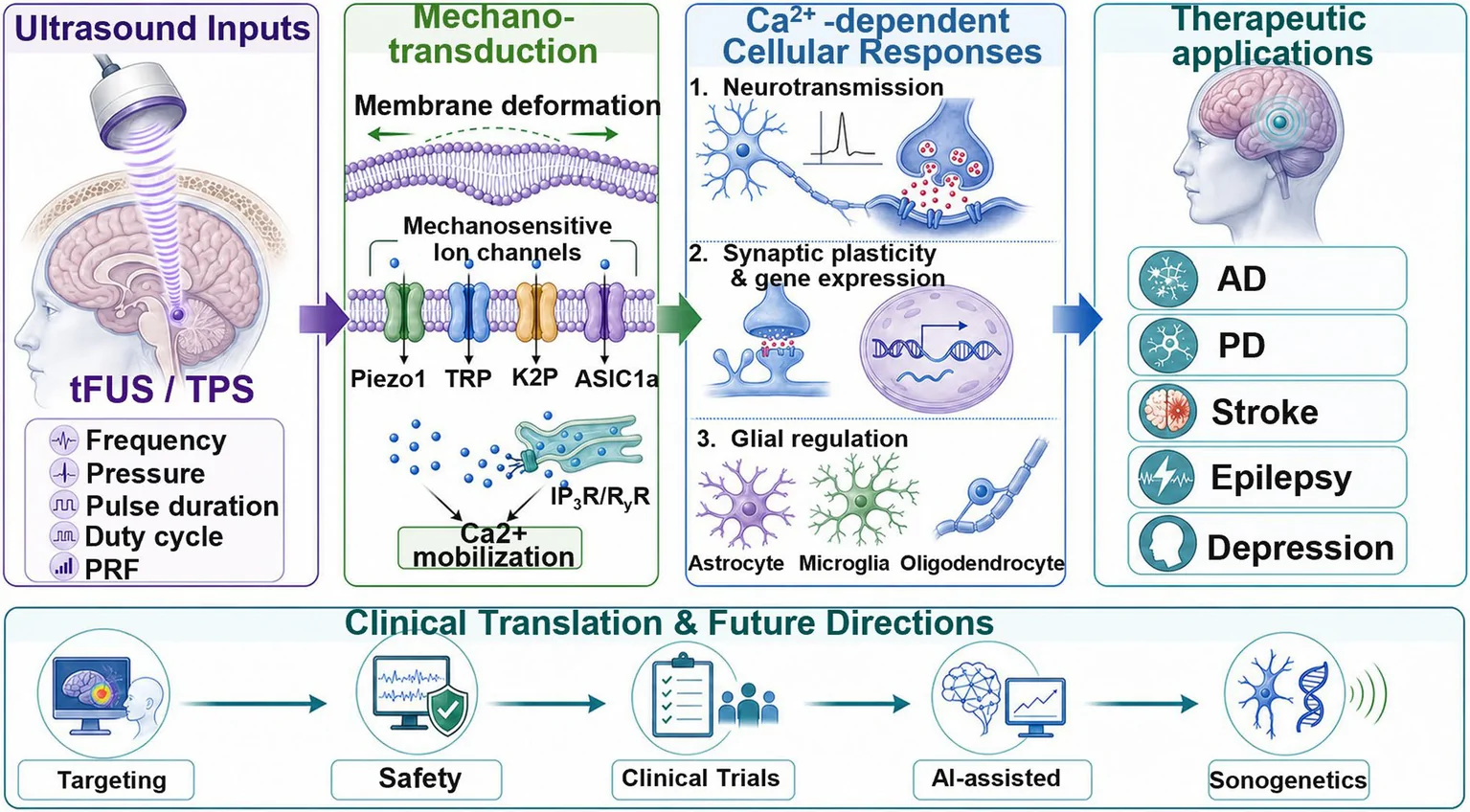
Acoustic inputs delivered using tFUS or TPS are defined by frequency, acoustic pressure, pulse duration, duty cycle, and PRF. Ultrasound-induced membrane deformation activates mechanosensitive ion channels, including Piezo1, TRP, K2P, and ASIC1a, while IP3R- and RyR-mediated release from intracellular stores contributes to Ca^2+^ mobilization. These signals regulate neurotransmission, synaptic plasticity and gene expression, and glial responses involving astrocytes, microglia, and oligodendrocytes. Their combined effects contribute to therapeutic responses in AD, PD, stroke, epilepsy, and depression. Clinical translation requires accurate targeting, rigorous safety assessment, standardized clinical trials, AI-assisted optimization, and further development of sonogenetic approaches. Abbreviations: AD, Alzheimer’s disease; AI, artificial intelligence; ASIC1a, acid-sensing ion channel 1a; Ca^2+^, calcium ion; IP3R, inositol 1,4,5-trisphosphate receptor; K2P, two-pore-domain potassium channel; PD, Parkinson’s disease; PRF, pulse repetition frequency; RyR, ryanodine receptor; tFUS, transcranial focused ultrasound; TPS, transcranial pulse stimulation; TRP, transient receptor potential.

## 1 Introduction

Brain disorders are among the most pressing global health challenges of the twenty-first century, imposing substantial medical, social, and economic burdens. According to the Global Burden of Disease (GBD) 2021 analysis, disorders affecting the nervous system were the leading cause of global health loss in 2021, affecting 3.40 billion people (43.1% of the global population) and accounting for approximately 443 million disability-adjusted life years (DALYs) and 11.1 million deaths. Between 1990 and 2021, global DALY counts attributable to these conditions increased by 18.2%, whereas the age-standardized DALY rate decreased by 27.0% (GBD 2021 Nervous System Disorders Collaborators, 2024). The Society for Fair Neuroscience (SEQUINS) states that more than 3 billion people worldwide are affected by neurological disorders (Ovbiagele, 2025), emphasizing the urgent need for innovative therapeutic interventions and long-term brain health strategies. Alzheimer’s disease (AD), Parkinson’s disease (PD), stroke, epilepsy, depression, and other psychiatric disorders are major contributors to this burden. Despite their distinct etiologies and clinical manifestations, these disorders commonly involve aberrant neural activity, synaptic dysfunction, neuroinflammation, neurovascular impairment, and disrupted brain networks. Current pharmacological therapies are often symptomatic, whereas surgical and conventional neuromodulation approaches are limited by invasiveness, inadequate spatial precision, restricted access to deep brain structures, or variable long-term efficacy. These limitations highlight the need for non-invasive technologies capable of precisely modulating neural circuits and multicellular brain processes.

### 1.1 Current neuromodulation technologies

Neuromodulation involves the targeted regulation of nervous system activity through electrical, magnetic, optical, chemical, or mechanical stimulation. Several neuromodulation technologies have entered clinical practice, each with distinct advantages and limitations (Figure 1). Deep brain stimulation (DBS) remains the most established invasive neuromodulation therapy and has demonstrated remarkable efficacy in Parkinson’s disease, essential tremor, dystonia, obsessive-compulsive disorder, and selected psychiatric disorders (Mahoney et al., 2022). However, DBS requires surgical implantation of intracranial electrodes and pulse generators, carrying risks of hemorrhage, infection, hardware malfunction, and high healthcare costs (Costin et al., 2025). Vagus nerve stimulation (VNS) has been approved for drug-resistant epilepsy and treatment-resistant depression (Jung et al., 2024). By stimulating afferent vagal pathways, VNS indirectly influences widespread cortical and subcortical networks involved in seizure regulation, cognition, and emotional processing (Chen and Liu, 2025). Nevertheless, surgical implantation remains necessary, and therapeutic effects often develop gradually over months. Among non-invasive approaches, transcranial magnetic stimulation (TMS) utilizes electromagnetic induction to non-invasively modulate cortical excitability and has become widely adopted for depression and neurorehabilitation (Luo et al., 2025). However, its penetration depth is generally limited to superficial cortical structures, restricting direct access to deep brain nuclei. Transcranial direct current stimulation (tDCS) delivers weak electrical currents through scalp electrodes to alter neuronal membrane excitability (Liu et al., 2018). Although tDCS is inexpensive, portable, and easy to administer, its diffuse current distribution and limited spatial specificity hinder precise targeting.

**Figure 1:**
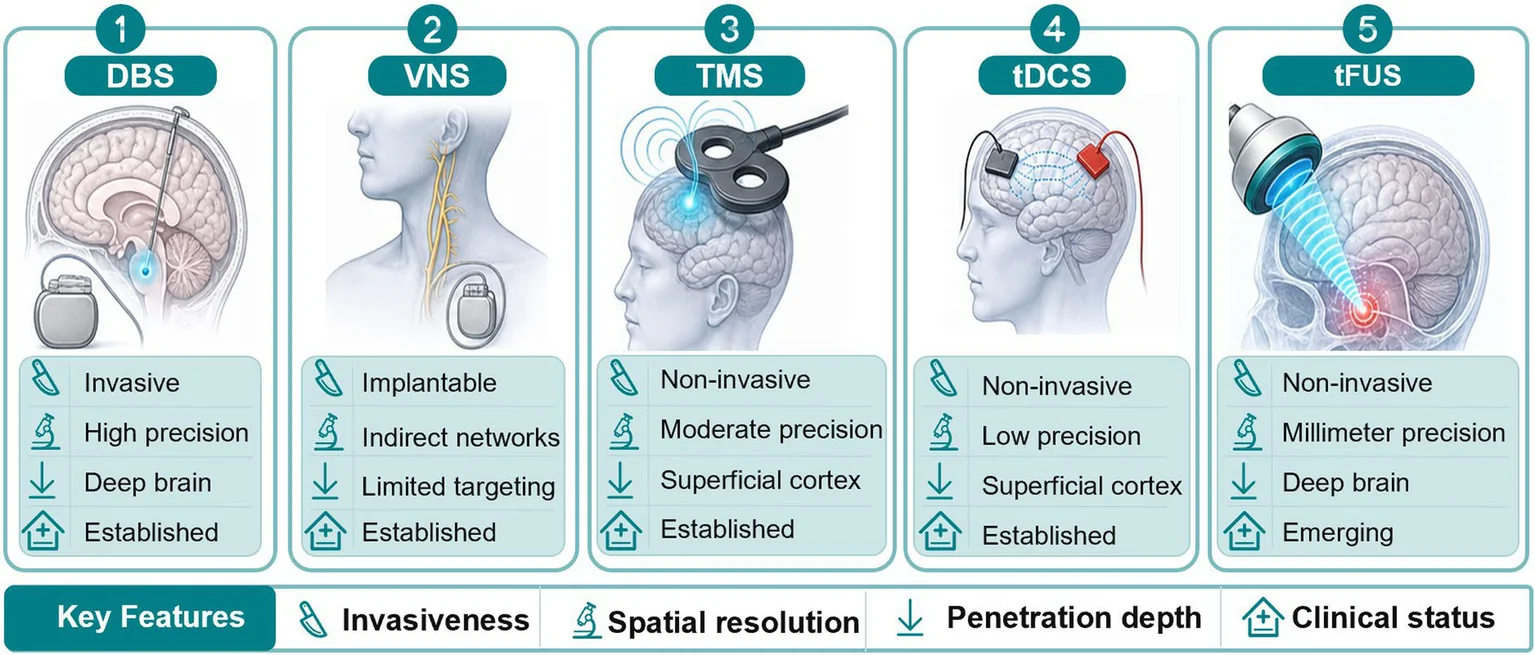
Comparison of current neuromodulation technologies. The technologies shown include deep brain stimulation (DBS), vagus nerve stimulation (VNS), transcranial magnetic stimulation (TMS), transcranial direct current stimulation (tDCS), and transcranial focused ultrasound (tFUS). These approaches are compared across key translational features, including invasiveness, spatial resolution, penetration depth and clinical status. DBS, deep brain stimulation; tDCS, transcranial direct current stimulation; tFUS, transcranial focused ultrasound; TMS, transcranial magnetic stimulation; VNS, vagus nerve stimulation.

In recent years, non-invasive ultrasound neuromodulation has emerged as a promising next-generation neuromodulation technology (Shi et al., 2025). In particular, low-intensity transcranial focused ultrasound (tFUS) can deliver focused acoustic energy through the intact skull to cortical and deep brain targets with millimeter-scale precision (Arulpragasam et al., 2026). Compared with electrical and magnetic stimulation, focused ultrasound combines deep penetration, high spatial resolution, reversibility, and extensive parameter tunability. Acoustic frequency, pressure, pulse repetition frequency, duty cycle, and sonication duration can be adjusted to produce distinct neuromodulatory effects. Compared with existing neuromodulation technologies, ultrasound possesses several distinctive advantages: (i) non-invasive transcranial delivery; (ii) superior spatial precision; (iii) accessibility to deep brain structures such as the hippocampus, thalamus, amygdala, and basal ganglia; (iv) flexible control of stimulation parameters; and (v) reversible modulation of neuronal and non-neuronal cellular processes. Collectively, these features position ultrasound as a potentially transformative technology for precision neuromodulation.

### 1.2 Scope of this review

This review provides an integrated account of ultrasound neuromodulation, linking acoustic exposure and dosimetry to cellular mechanotransduction, disease-oriented evidence, and clinical translation. The primary focus is non-ablative ultrasound neuromodulation, including low-intensity transcranial focused ultrasound and transcranial pulse stimulation. Particular attention is given to the combined effects of acoustic parameters, direct mechanical interactions with neural tissue, mechanosensitive ion channels, calcium-dependent regulation of neuronal function and synaptic plasticity, and glial responses. Evidence for therapeutic applications in Alzheimer’s disease (AD), Parkinson’s disease (PD), stroke, epilepsy and depression is critically evaluated alongside the current clinical evidence, exposure-related safety considerations, and limitations in efficacy and durability. The review also addresses unresolved mechanistic questions, skull-related acoustic variability, dosimetric and reporting standardization, and emerging approaches such as closed-loop stimulation, artificial intelligence-assisted optimization, and sonogenetics. Thermal ablation and microbubble-assisted blood–brain barrier (BBB) opening are addressed selectively for contextual comparison in disease-oriented, safety, and translational discussions and are treated as mechanistically and therapeutically distinct from non-ablative neuromodulation.

## 2 Fundamentals of ultrasound neuromodulation

Unlike conventional neuromodulation technologies that rely on electrical or magnetic stimulation, ultrasound employs mechanical acoustic waves to interact with neural tissues. Through precise manipulation of acoustic parameters, ultrasound can non-invasively modulate neuronal excitability, alter network connectivity, and influence cellular signaling pathways within both superficial and deep brain regions (Rabut et al., 2020). Neuromodulatory efficacy and safety depend on the combined effects of acoustic frequency, pressure, pulse structure, and sonication duration (Quarato et al., 2023).

### 2.1 Ultrasound physics and dosimetry

Ultrasound consists of mechanical pressure waves above the upper limit of human hearing (>20 kHz) and propagates through tissue as alternating compression and rarefaction cycles (Kim et al., 2024a). In transcranial neuromodulation, its interaction with brain tissue is governed primarily by acoustic frequency, pressure and intensity, pulse duration, duty cycle, pulse repetition frequency (PRF), and total sonication time (Nandi et al., 2025; Cheng et al., 2026).

Frequency determines the balance between skull penetration and focal precision: lower frequencies, commonly 200–700 kHz, undergo less skull attenuation and are therefore suitable for deep targets, whereas higher frequencies produce smaller focal volumes but greater attenuation and phase distortion (Lee et al., 2024; Blackmore et al., 2019; Demene et al., 2021). Acoustic exposure should be reported using pressure- and intensity-based metrics, particularly peak negative pressure, spatial-peak pulse-average intensity (I_SPPA), and spatial-peak temporal-average intensity (I_SPTA) (Keihani et al., 2024; Zhang T. et al., 2021). Pressure and I_SPPA primarily reflect transient mechanical stimulation, whereas I_SPTA incorporates temporal energy deposition and is more closely related to cumulative heating (Arulpragasam et al., 2026; Cox et al., 2025; Moyano et al., 2022). Pulsed protocols are further defined by pulse duration, duty cycle, and PRF (Fomenko et al., 2020; Kim et al., 2014; Yang et al., 2025; Zhang et al., 2023; Sherman et al., 2024; Nowacka et al., 2025). These parameters are interdependent. Identical intensity values may produce different biological effects when delivered using different frequencies or temporal patterns. Moreover, skull thickness, composition, incidence angle, and target depth alter the pressure reaching the brain. Accordingly, rigorous dosimetry should distinguish free-field from estimated *in situ* exposure and report transducer geometry, waveform, coupling conditions, skull-correction procedures, and thermal or cavitation monitoring when applicable. Standardized reporting of these variables is essential for comparing studies, reproducing neuromodulatory effects, and defining safe and effective exposure window.

### 2.2 Major transcranial ultrasound technologies

Non-ablative transcranial ultrasound neuromodulation encompasses focused approaches that can access cortical and subcortical targets through the intact skull. Low-intensity focused ultrasound (LIFU) uses focused acoustic energy at exposure levels intended to modulate neural function without producing thermal tissue destruction. When delivered transcranially, this approach is commonly implemented as low-intensity transcranial focused ultrasound (tFUS). Conventional low-intensity tFUS protocols generally employ multicycle acoustic pulses organized into pulse trains, whose biological effects can be adjusted through carrier frequency, acoustic pressure, pulse duration, pulse repetition frequency, duty cycle, and sonication duration. Transcranial pulse stimulation (TPS) extends non-ablative transcranial ultrasound neuromodulation through a different temporal delivery pattern based on isolated ultrashort acoustic pulses (Figure 2).

**Figure 2:**
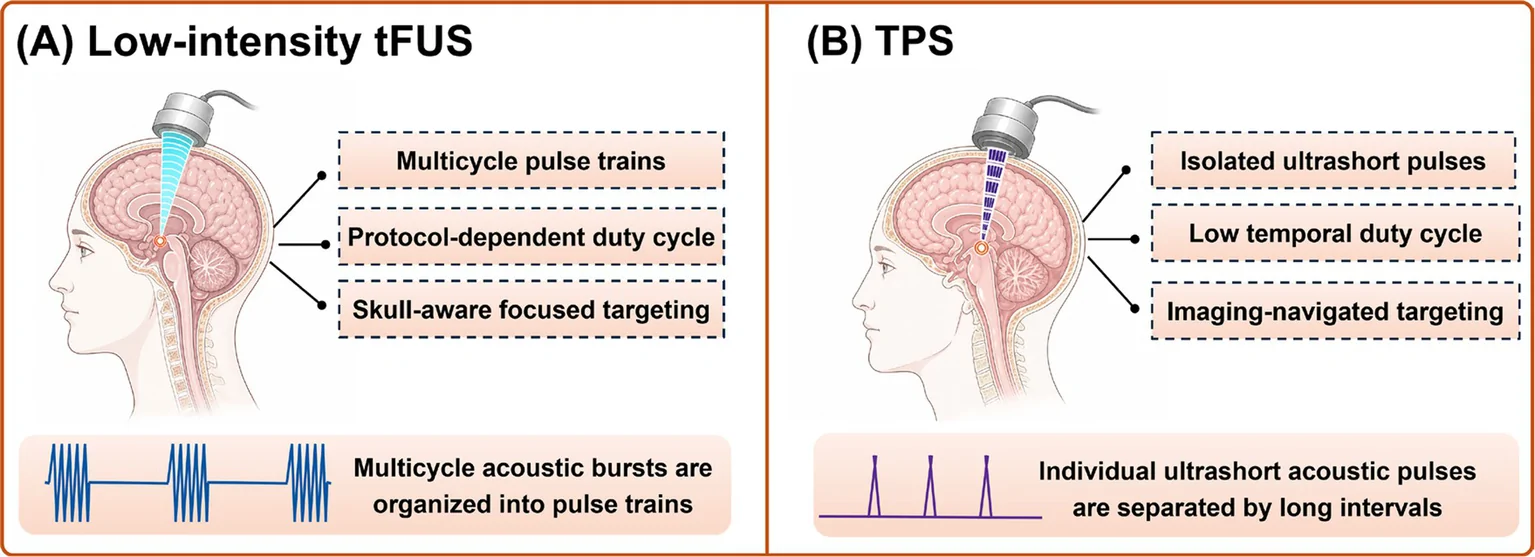
Principal paradigms of non-ablative transcranial ultrasound neuromodulation. **(A)** Low-intensity tFUS delivers multicycle acoustic bursts organized into pulse trains through the intact skull to a focused intracranial target. Its temporal duty cycle is protocol-dependent, and skull-aware treatment planning may be required to compensate for skull-induced attenuation and phase aberration. **(B)** TPS delivers isolated ultrashort acoustic pulses separated by relatively long intervals, resulting in a low temporal duty cycle. Imaging-based navigation is commonly used to guide pulse delivery to predefined brain targets. Both approaches provide targeted, transcranial, and non-ablative stimulation but differ principally in their temporal pulse structure and delivery paradigm. tFUS, transcranial focused ultrasound; TPS, transcranial pulse stimulation.

#### 2.2.1 Low-intensity tFUS

By concentrating low-intensity acoustic energy within a millimeter-scale focal volume, low-intensity tFUS can modulate neuronal activity while minimizing thermal accumulation and tissue damage. Experimental studies have shown that low-intensity focused ultrasound can alter neuronal firing, neurotransmitter signaling, synaptic plasticity, and network activity through mechanically mediated cellular response (Puppalla et al., 2026). Target engagement and functional modulation have been demonstrated in rodents, non-human primates, and humans (Ho et al., 2025; Darrow, 2019; Cox et al., 2025), encompassing cortical and subcortical structures such as the primary motor and somatosensory cortices, thalamus, hippocampus, amygdala, and basal ganglia (Fomenko et al., 2018). Skull-dependent attenuation and phase aberration can displace or weaken the intracranial focus, necessitating individualized acoustic modeling and image-guided targeting (Li Z. et al., 2025; Wang et al., 2026). Clinical studies indicate that appropriately controlled low-intensity tFUS can reversibly modulate sensory, motor, cognitive, and affective circuits, although the magnitude and direction of the responses remain dependent on acoustic parameters, anatomical targets, and individual skull characteristics (Kuhn et al., 2023).

#### 2.2.2 TPS

TPS is a clinically oriented ultrasound platform that delivers individual ultrashort acoustic pulses rather than conventional multicycle pulse trains. Its low temporal duty cycle limits cumulative energy deposition and thermal effects while retaining transient mechanical stimulation. TPS is typically combined with magnetic resonance imaging-based navigation, allowing acoustic pulses to be delivered to predefined cortical and subcortical targets. Preliminary clinical studies in patients with AD have reported improvements in cognitive performance and functional connectivity within memory-related networks following repeated TPS treatment (Chen et al., 2024; Truong et al., 2022). However, current evidence is derived mainly from exploratory studies, and larger sham-controlled trials are required to determine its efficacy, optimal dosing, and durability.

### 2.3 Neuromodulation parameters and biological responses

Unlike pharmacological interventions that primarily depend on drug concentration and receptor interactions, ultrasound-induced biological effects can be dynamically adjusted through multiple acoustic parameters (Figure 3). Neuromodulatory outcomes are highly sensitive to stimulation settings, and even subtle changes in acoustic parameters may lead to markedly different physiological responses, ranging from neuronal excitation and inhibition to long-term synaptic plasticity and network reorganization (Li Z. et al., 2025; Blackmore et al., 2019). Importantly, there is currently no universally accepted stimulation protocol for ultrasound neuromodulation. Instead, biological responses arise from complex interactions among ultrasound parameters, tissue properties, target brain regions, and cellular mechanotransduction mechanisms.

**Figure 3:**
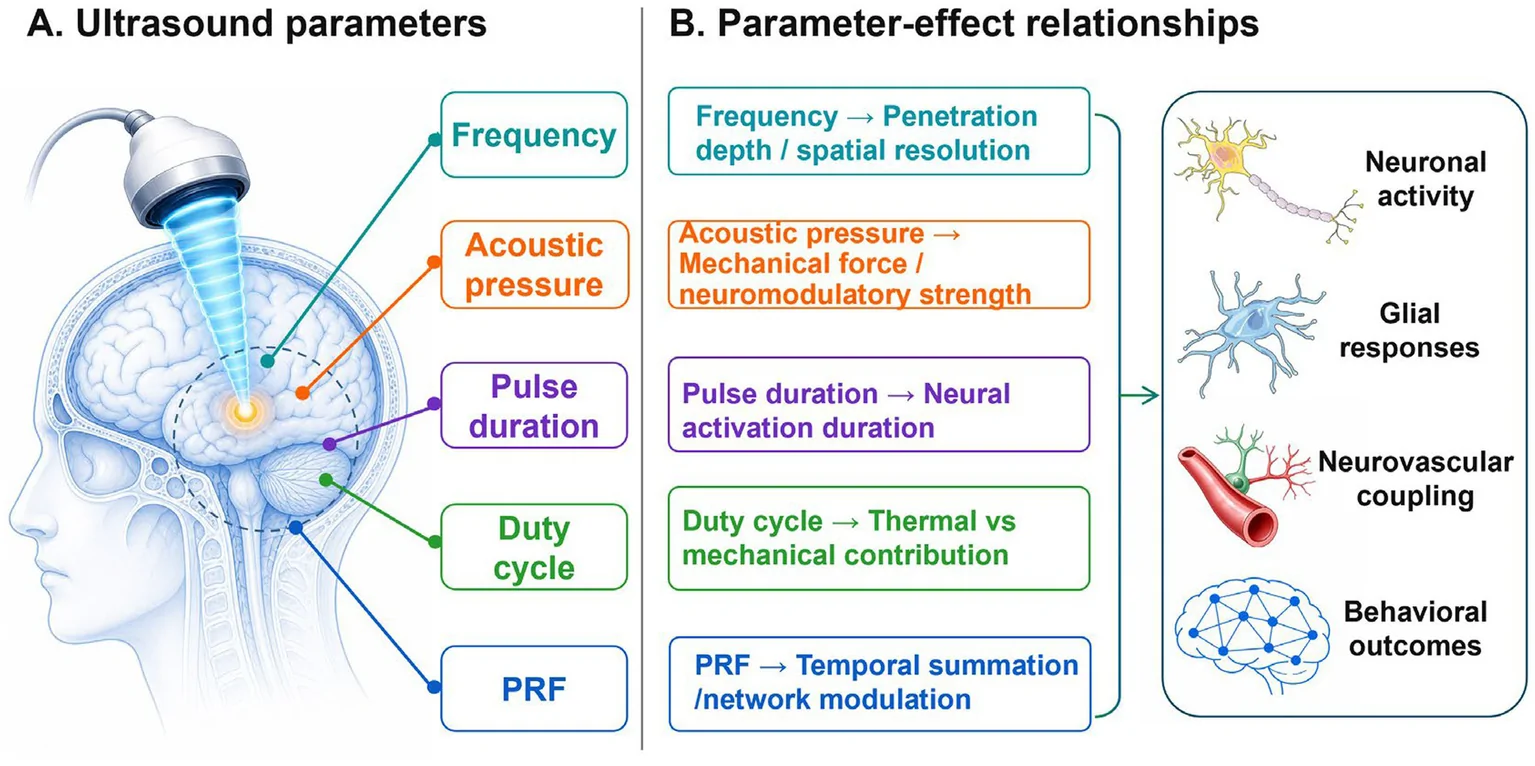
Parameter-effect relationships in ultrasound neuromodulation. **(A)** Ultrasound exposure is characterized by interacting acoustic parameters, including frequency, acoustic pressure, pulse duration, duty cycle, and PRF. Frequency influences transcranial penetration and spatial resolution. Acoustic pressure determines the magnitude of mechanical force. Pulse duration regulates the duration of individual acoustic exposures. Duty cycle affects time-averaged energy deposition, and PRF determines the temporal pattern of pulse delivery. **(B)** The combined effects of these parameters regulate neuronal activity, glial responses, neurovascular coupling, and behavioral outcomes. Optimization must therefore consider the complete acoustic exposure rather than treating individual parameters as independent predictors of neuromodulatory efficacy or safety. PRF, pulse repetition frequency.

#### 2.3.1 Frequency-effect relationship

Frequency is one of the most fundamental determinants of ultrasound propagation and neuromodulatory efficacy. It influences skull penetration, focal volume, spatial resolution, energy attenuation, and the magnitude of mechanical forces delivered to neural tissue. Generally, lower frequencies (<700 kHz) exhibit superior transcranial penetration because they undergo less attenuation and phase distortion when passing through the skull (Lee and Chang, 2026). Consequently, frequencies ranging from 250 to 700 kHz have become the most commonly employed range in contemporary low-intensity tFUS studies (Fomenko et al., 2018; Wang et al., 2019). These frequencies allow acoustic energy to reach deep brain structures such as the hippocampus, thalamus, basal ganglia, and amygdala while maintaining acceptable focal precision. In contrast, higher frequencies (>1 MHz) provide smaller focal zones and improved spatial specificity (Wang et al., 2019). This enhanced targeting capability is advantageous when selective modulation of small cortical or subcortical structures is desired. However, increased attenuation may substantially reduce acoustic transmission efficiency through the skull, thereby limiting their applicability in deep-brain neuromodulation.

Beyond physical propagation characteristics, frequency may directly influence cellular mechanotransduction. Recent studies suggest that mechanosensitive ion channels, including Piezo1, TRPA1, TRPV4, and TREK-family potassium channels, exhibit differential sensitivities to acoustic stimulation. Variations in frequency may therefore alter channel activation probability, intracellular calcium signaling, and neuronal firing patterns (Liu et al., 2025; Nims et al., 2024).

#### 2.3.2 Acoustic pressure-effect relationship

Acoustic pressure represents the peak mechanical force generated by ultrasound waves and is considered one of the most influential determinants of biological efficacy. During ultrasound propagation, alternating positive and negative pressure cycles induce mechanical stress on cell membranes, extracellular matrices, and intracellular structures (Yoo et al., 2025). This mechanical perturbation is thought to initiate many of the mechanotransduction processes underlying ultrasound neuromodulation. Experimental studies have demonstrated pressure-dependent increases in neuronal firing rates, cortical evoked potentials, and behavioral responses across multiple animal models (Song et al., 2023; He et al., 2025). However, the relationship between acoustic pressure and biological outcome is not strictly linear. Ultrasound below a target- and protocol-specific threshold may fail to produce a measurable response, whereas increasing pressure beyond this threshold may strengthen neuromodulation but also increase the risks of tissue heating, inertial cavitation, vascular injury, and BBB perturbation. Moreover, whether ultrasound produces excitation or inhibition cannot be predicted from acoustic pressure alone, because response polarity also depends on carrier frequency, pulse structure, target region, cell type, and baseline neural activity (Li F. et al., 2025; He et al., 2025). Acoustic pressure should therefore be interpreted as one component of the complete exposure protocol rather than as an independent determinant of response magnitude or direction.

#### 2.3.3 Pulse duration and duty cycle-effect relationship

Pulse duration and duty cycle jointly define the temporal pattern and time-averaged exposure of ultrasound stimulation. Pulse duration is the duration of each acoustic pulse, whereas duty cycle is the fraction of time during which ultrasound is transmitted (Li F. et al., 2025). Their effects cannot be interpreted independently of pulse repetition frequency, acoustic pressure, carrier frequency, and total sonication duration. Tone-burst duration also influences stimulation efficacy, although the relationship is not simply monotonic. Kim et al. reported that tone-burst durations of 1–5 ms elicited motor responses at lower threshold acoustic intensities than 0.25–0.5 ms bursts in anesthetized rats (Kim et al., 2014). Duty cycle influences temporal-average intensity and thermal accumulation only in relation to the remaining exposure variables. Retrospective thermal simulations of seven rodent neuromodulation protocols estimated brain temperature rises of 0.002–0.8 °C for most settings but approximately 7 °C for one high-exposure protocol, demonstrating that a low nominal duty cycle alone does not establish a non-thermal mechanism (Constans et al., 2018). Therefore, pulse duration and duty cycle should be reported and interpreted as components of the complete acoustic exposure rather than as independent predictors of excitation, inhibition, or safety.

#### 2.3.4 Pulse repetition frequency-effect relationship

Pulse repetition frequency (PRF) determines how frequently acoustic pulses are delivered during stimulation and therefore shapes the temporal pattern of neural activation. Although PRF does not directly affect ultrasound propagation through tissue, it significantly influences neuronal temporal summation, calcium dynamics, synaptic transmission, and network synchronization (Sherman et al., 2024). Yu et al. (2021) delivered 500-kHz tFUS to the primary somatosensory cortex of isoflurane-anesthetized rats and compared PRFs of 30, 300, 1,500, 3,000, and 4,500 Hz using intracranial single-unit recordings. When duty cycle was held constant, the normalized firing rates of 174 regular-spiking units differed significantly across PRFs, and a significant interaction was observed between PRF and neuronal type. Ramachandran et al. (2025) subsequently examined the same five PRFs in the somatosensory cortex of awake head-fixed rats using 1.5-MHz tFUS, a 200-μs tone-burst duration, and a 67-ms sonication duration. PRF significantly affected the time-locked firing of regular-spiking units, with 1,500–4,500 Hz producing stronger responses than 30 or 300 Hz. Because duty cycle and temporal-average intensity increased with PRF in the latter experiment, these findings describe the effects of the complete pulse configuration rather than an isolated effect of PRF. Collectively, these results indicate that PRF can influence the timing and cell-type distribution of ultrasound-evoked neuronal firing.

#### 2.3.5 Parameter interactions and neuromodulatory outcomes

Although individual acoustic parameters are often studied independently, biological responses are ultimately determined by their combined interactions. Frequency, acoustic pressure, pulse duration, duty cycle, and PRF collectively define the acoustic dose delivered to neural tissue and therefore shape the magnitude and nature of neuromodulatory effects. For example, increasing acoustic pressure may enhance neuromodulatory efficacy under low-duty-cycle conditions but produce excessive energy deposition when combined with prolonged pulse durations (Nandi et al., 2025). Similarly, a frequency that is effective for cortical stimulation may be suboptimal for deep-brain targeting because of differences in skull attenuation and focal geometry. Recent computational models suggest that different parameter combinations may preferentially activate distinct mechanotransduction pathways. Certain stimulation paradigms appear to primarily influence neuronal membranes, whereas others more strongly affect astrocytic calcium signaling, microglial activation, or neurovascular responses (Chen et al., 2025). Consequently, ultrasound neuromodulation should increasingly be viewed as a multidimensional parameter space rather than a single stimulation modality.

## 3 Cellular and molecular mechanisms of ultrasound neuromodulation

Unlike electrical stimulation, which directly alters membrane potentials through externally applied currents, ultrasound interacts with neural tissues primarily through mechanical forces (Figure 4). These forces initiate a cascade of mechanotransduction events that span multiple biological scales, from membrane deformation and ion channel activation to network-level modulation and behavioral responses (Kamimura et al., 2020; Jerusalem et al., 2019). Ultrasound-induced neuromodulation cannot be attributed to a single mechanism. Instead, it likely involves the coordinated contribution of neuronal membranes, mechanosensitive ion channels, intracellular calcium signaling, glial cells, vascular components, and extracellular matrix structures. The relative importance of these mechanisms may vary according to stimulation parameters, target brain regions, and physiological conditions. Current mechanistic models generally propose a hierarchical pathway in which acoustic forces are first converted into cellular mechanical stimuli, subsequently triggering biochemical signaling pathways that ultimately regulate neuronal excitability and circuit function.

**Figure 4:**
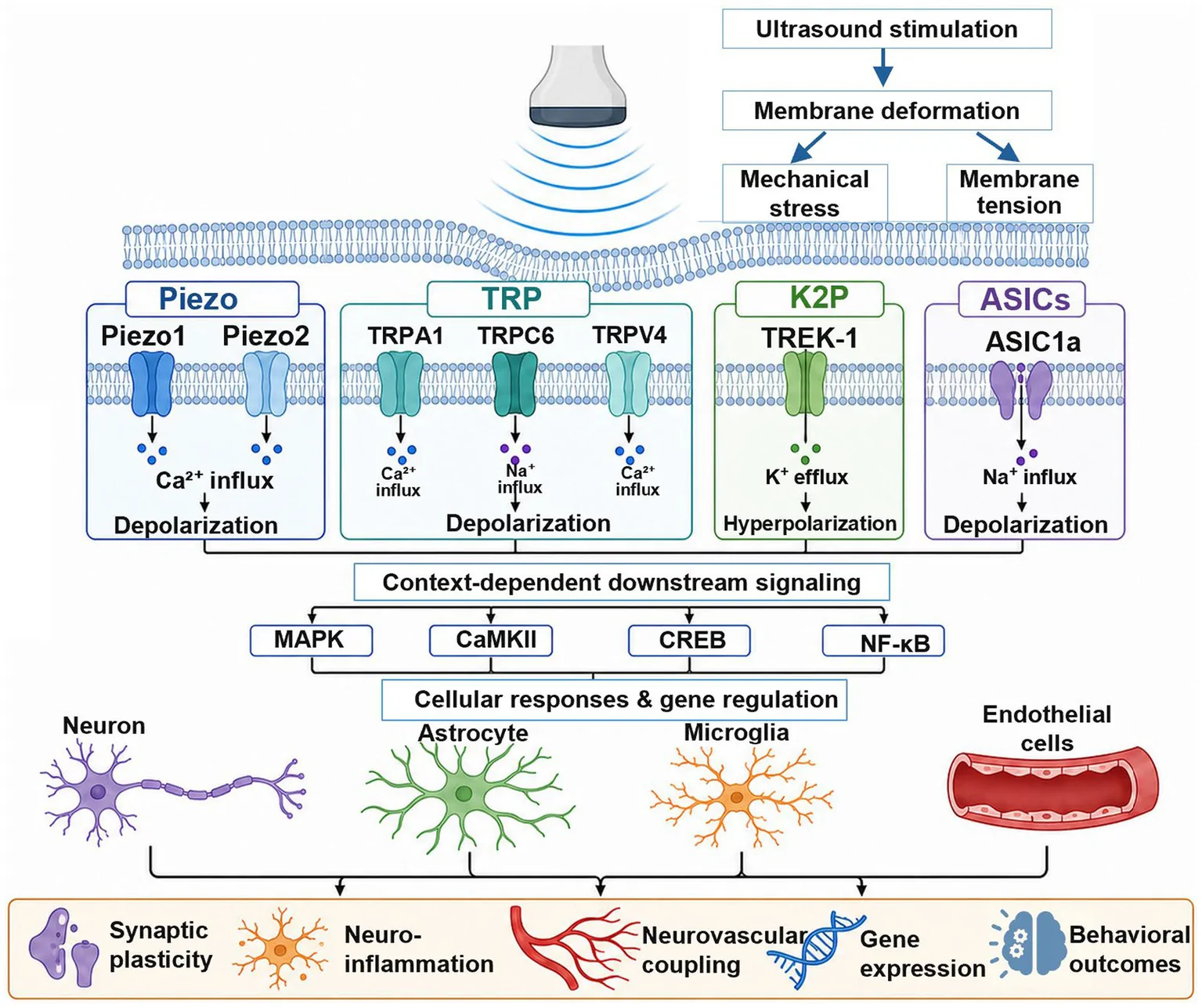
Mechanotransduction pathways underlying ultrasound neuromodulation. Ultrasound stimulation deforms the plasma membrane and generates mechanical stress and membrane tension, thereby activating multiple classes of mechanosensitive ion channels. Piezo channels promote Ca^2+^ influx and membrane depolarization, whereas TRP channels, including TRPA1, TRPC6, and TRPV4, mediate Ca^2+^ and Na^+^ influx. ASICs, exemplified by ASIC1a, contribute to Na^+^ influx and depolarization, whereas K2P channels such as TREK-1 facilitate K^+^ efflux and membrane hyperpolarization. These ion channel-dependent signals converge on downstream pathways involving MAPK, CaMKII, CREB, and NF-κB. Across neurons, astrocytes, microglia, and endothelial cells, these pathways regulate synaptic plasticity, neuroinflammation, neurovascular coupling, gene expression, and behavioral outcomes. ASIC, acid-sensing ion channel; ASIC1a, acid-sensing ion channel 1a; Ca^2+^, calcium ion; CaMKII, calcium/calmodulin-dependent protein kinase II; CREB, cyclic adenosine monophosphate response element-binding protein; K2P, two-pore-domain potassium channel; K^+^, potassium ion; MAPK, mitogen-activated protein kinase; Na^+^, sodium ion; NF-κB, nuclear factor kappa B; TREK-1, TWIK-related potassium channel 1; TRP, transient receptor potential; TRPA1, transient receptor potential ankyrin 1; TRPV4, transient receptor potential vanilloid 4; TRPC6, transient receptor potential canonical 6.

### 3.1 Direct mechanical effects

The most widely accepted mechanism underlying ultrasound neuromodulation is based on the direct mechanical interaction between acoustic waves and biological tissues. As ultrasound propagates through the brain, oscillating pressure fields generate repetitive cycles of compression and rarefaction that exert mechanical forces on cellular structures. Unlike high-intensity therapeutic ultrasound used for tissue ablation, low-intensity ultrasound neuromodulation primarily produces non-thermal mechanical effects, which are thought to represent the initial trigger for downstream biological responses.

#### 3.1.1 Acoustic radiation force

One of the principal mechanical effects generated by ultrasound is acoustic radiation force (ARF). ARF arises from momentum transfer between propagating ultrasound waves and biological tissues, producing a net force capable of inducing microscopic tissue displacement. Experimental measurements have demonstrated that focused ultrasound can generate tissue displacements ranging from nanometers to several micrometers depending on stimulation parameters and tissue properties (Lee et al., 2020; Li and Liu, 2025). Although these displacements are small, they occur at extremely high temporal frequencies and are sufficient to deform neuronal membranes and alter mechanosensitive cellular structures. Several computational studies have suggested that acoustic radiation force may represent the dominant mechanism responsible for neuromodulation under low-intensity stimulation conditions (Kim et al., 2023; Menz et al., 2019). By inducing localized membrane strain, ARF can alter membrane tension and subsequently influence the gating behavior of mechanosensitive ion channels. Importantly, the magnitude of radiation force increases with acoustic intensity and is influenced by tissue density, acoustic absorption, and focal geometry.

#### 3.1.2 Membrane deformation and mechanical stress

Cell membranes constitute the primary interface between ultrasound waves and neural cells. As acoustic forces propagate through tissue, transient membrane deformation occurs as a result of localized pressure gradients and mechanical displacement. Neuronal plasma membranes are highly sensitive mechanical structures containing numerous mechanosensitive proteins embedded within lipid bilayers. Ultrasound-induced membrane deformation can alter membrane curvature, lipid organization, and cytoskeletal interactions (Gianoli et al., 2017). Such changes influence the gating of mechanosensitive ion channels and thereby modify membrane conductance and neuronal excitability. Supporting this general mechanotransduction principle, Kanda et al. demonstrated that mechanical stretching of peripheral axons suppressed action-potential propagation (Kanda et al., 2023). Although this experiment did not involve ultrasound, it provides direct evidence that membrane tension can regulate neuronal excitability. In ultrasound neuromodulation, membrane deformation therefore represents a plausible physical link between acoustic forces and ion-channel gating.

#### 3.1.3 Intramembrane cavitation hypothesis

Different from classical cavitation involving microbubble formation in extracellular fluids, intramembrane cavitation refers to the formation of nanoscale gaseous cavities within the hydrophobic core of lipid bilayers (Krasovitski et al., 2011). According to this hypothesis, oscillatory acoustic pressure causes repeated expansion and contraction of these nanocavities, resulting in dynamic alterations in membrane capacitance and transmembrane voltage (Plaksin et al., 2016). These capacitance fluctuations may generate displacement currents capable of influencing neuronal membrane potential and action potential generation. Computational simulations suggest that intramembrane cavitation can occur under acoustic conditions commonly employed in ultrasound neuromodulation experiments (Lemaire et al., 2021). Although direct experimental evidence remains limited, the intramembrane cavitation model has become one of the most influential theoretical frameworks for understanding ultrasound-induced neural activation.

#### 3.1.4 Cytoskeletal mechanotransduction

Beyond membrane deformation, ultrasound-induced mechanical forces may be transmitted throughout the cell via the cytoskeleton. The neuronal cytoskeleton, consisting primarily of actin filaments, microtubules, and intermediate filaments, serves as an essential structural network for mechanotransduction. Mechanical perturbations applied to the plasma membrane can rapidly propagate through cytoskeletal elements to intracellular organelles, including the nucleus, endoplasmic reticulum, and mitochondria. This mechanical signaling influences intracellular calcium dynamics, gene expression, mitochondrial function, and synaptic activity. Recent evidence indicates that mechanosensitive proteins are frequently associated with cytoskeletal structures, suggesting that cytoskeletal integrity may be required for efficient ultrasound-induced neuromodulation (Chu P. C. et al., 2022).

#### 3.1.5 Mechanical regulation of synaptic function

Mechanical forces generated by ultrasound may also directly influence synaptic physiology. Synaptic membranes contain numerous mechanosensitive components involved in neurotransmitter release, vesicle trafficking, receptor organization, and cytoskeletal remodeling. Experimental studies have demonstrated that ultrasound stimulation can alter presynaptic vesicle release probability and modulate postsynaptic receptor activity (Weinreb and Moses, 2022; Shi et al., 2025). In addition, repeated transcranial LIPUS delivered to the hippocampal CA1 region increased dendritic spine density, spontaneous excitatory postsynaptic current frequency, and GluN2A expression in rats, providing ultrasound-specific evidence for structural and functional synaptic plasticity (Huang et al., 2019). These effects are particularly relevant because long-term alterations in synaptic strength constitute the cellular basis of learning, memory, and functional recovery following neurological injury (Sorum et al., 2023; Del Rosario-Gilabert et al., 2024).

### 3.2 Mechanosensitive ion channels

Mechanosensitive ion channels provide a molecular interface through which ultrasound-induced membrane deformation and mechanical stress can be converted into ionic currents and intracellular signaling. Their activation can alter membrane potential, intracellular Ca^2+^ concentration, neurotransmitter release, and downstream transcriptional programs (Del Rosario-Gilabert et al., 2024; Qiu et al., 2019). However, the channels implicated in ultrasound neuromodulation are supported by different levels of evidence. Direct evidence requires ultrasound-evoked responses to be attenuated by pharmacological inhibition, genetic knockdown, or channel deletion in native cells or tissues. In contrast, general mechanosensitivity, computational predictions, and enhanced ultrasound responsiveness after heterologous overexpression provide supportive but less definitive evidence. Current findings provide strong ultrasound-specific evidence for Piezo1, TRPA1, TRPC6, ASIC1a, and selected two-pore-domain potassium channels, although their relative contributions remain dependent on cell type, brain region, and acoustic protocol (Yoo et al., 2022; Song et al., 2023).

#### 3.2.1 Piezo1

Piezo1 is a mechanically activated, non-selective cation channel with a trimeric propeller-like structure that is highly sensitive to membrane curvature and tension (Coste et al., 2010; Ge et al., 2015; Lin et al., 2019). Channel opening permits Ca^2+^ and Na^+^ influx, thereby coupling mechanical deformation to membrane depolarization and calcium-dependent signaling. Direct *in vitro* evidence supports its involvement in ultrasound responsiveness: low-intensity ultrasound induced Ca^2+^ elevations and neuronal activation that were attenuated by pharmacological inhibition or knockdown of Piezo1 (Qiu et al., 2019). These findings provide a causal link between ultrasound exposure and Piezo1-dependent signaling in cultured cells.

Nevertheless, evidence from native brain tissue remains more limited. Piezo1 is expressed in neurons, astrocytes, microglia, endothelial cells, and neural progenitors, and it regulates processes such as microglial amyloid-β clearance (Jantti et al., 2022). Its broader involvement in pain and mechano-inflammatory signaling further supports its biological relevance within the nervous system. However, these observations demonstrate the physiological functions of Piezo1 rather than its specific contribution to ultrasound neuromodulation. Similarly, enhanced ultrasound sensitivity following channel overexpression supports its potential use in sonogenetics but does not establish that endogenous Piezo1 is the dominant transducer under physiological conditions. Further cell-type-specific knockout and *in vivo* electrophysiological studies are therefore required to define its contribution across brain regions and acoustic protocols.

#### 3.2.2 TRPA1

Transient receptor potential ankyrin 1 (TRPA1) is a non-selective cation channel responsive to mechanical, chemical, and oxidative stimuli (Hu Z. et al., 2023). Its activation produces Ca^2+^ influx and membrane depolarization, making it a plausible mediator of ultrasound-induced excitation. Among the TRP channels, TRPA1 is supported by relatively strong ultrasound-specific evidence. Low-intensity ultrasound has been shown to activate astrocytic TRPA1, inducing Ca^2+^ elevations and gliotransmitter release that subsequently modulate neuronal activity (Oh et al., 2019). This finding indicates that ultrasound may influence neural circuits indirectly through astrocyte-dependent signaling rather than exclusively through direct neuronal stimulation.

TRPA1 has also been investigated as a sonogenetic actuator. Heterologous expression of human TRPA1 substantially enhanced ultrasound-evoked Ca^2+^ responses and membrane currents, whereas pharmacological inhibition attenuated these effects (Duque et al., 2022). Although this provides compelling evidence that TRPA1 can confer ultrasound sensitivity, overexpression-based sonogenetic responses should be distinguished from the physiological contribution of endogenous TRPA1. Its native expression level, cellular distribution, and activation threshold may differ substantially across brain regions. Thus, TRPA1 is a well-supported ultrasound-responsive channel, but its relative contribution to neuronal and glial responses in the intact human brain remains uncertain.

#### 3.2.3 TRPC6

Transient receptor potential canonical 6 (TRPC6) is a mechanosensitive, non-selective cation channel expressed in cortical and hippocampal neurons. Matsushita et al. demonstrated that ultrasound elicited rapid Ca^2+^ transients through extracellular Ca^2+^ entry in cultured mouse cortical and hippocampal neurons. Pharmacological inhibition or genetic deletion of TRPC6 almost abolished these responses, whereas lentiviral re-expression of TRPC6 in knockout neurons restored ultrasound responsiveness. These experiments provide direct evidence that endogenous TRPC6 contributes to neuronal ultrasound sensing (Matsushita et al., 2024). This study further showed that transcranial ultrasound elicited intensity-dependent cortical population activity in anesthetized mice, whereas intracerebroventricular administration of the TRPC6 antagonist BI-749327 significantly attenuated the evoked neuronal firing (Matsushita et al., 2024). These findings identify TRPC6 as an important mediator of ultrasound-induced neuronal activation in the mouse brain.

#### 3.2.4 TREK-1, TRAAK, and other K2P channels

Two-pore-domain potassium channels (K2P) differ functionally from Piezo and TRP channels because their activation generally promotes K^+^ efflux, membrane hyperpolarization, and reduced neuronal excitability. TREK-1 and TRAAK are directly gated by membrane tension and lipid bilayer deformation (Sonkusare et al., 2012; Shibasaki et al., 2014; Iadecola, 2017; Brohawn et al., 2014) providing a plausible mechanism for ultrasound-induced inhibition. Direct ultrasound evidence is currently stronger for TRAAK than for TREK-1. Ultrasound has been shown to activate TRAAK through increased membrane tension, producing outward K^+^ currents without requiring an additional intracellular signaling pathway (Sorum et al., 2021). These findings suggest that K2P channel recruitment may contribute to inhibitory neuromodulation. However, the extent to which endogenous TREK-1 or TRAAK determines ultrasound-induced inhibition in native neural circuits remains unresolved. Their effects are likely influenced by channel expression, membrane lipid composition, acoustic parameters, and the simultaneous activation of excitatory mechanosensitive channels.

#### 3.2.5 Acid-sensing ion channels (ASICs)

Piezo2, TRPV4, and acid-sensing ion channels (ASICs) have also been implicated in ultrasound responsiveness, although the supporting evidence differs substantially. Piezo2 is highly sensitive to mechanical deformation and is essential for mechanosensation and proprioception. However, direct evidence that endogenous Piezo2 mediates ultrasound responses in central neural circuits remains scarce. Its proposed role is based primarily on its established mechanosensitivity and expression in selected sensory neuronal populations. TRPV4 responds to membrane stretching, osmotic stress, fluid shear, and other mechanical stimuli. It regulates Ca^2+^ signaling in endothelial cells and astrocytes and contributes to vascular function, gliotransmission, and neurovascular interactions (Shibasaki et al., 2014; Iadecola, 2017). These properties make TRPV4 a plausible mediator of multicellular ultrasound responses. Nevertheless, most available evidence concerns general mechanical or osmotic stimulation rather than direct ultrasound exposure. Its role in ultrasound neuromodulation should therefore be considered provisional until channel-specific inhibition or deletion is shown to alter ultrasound-evoked responses in native neural tissue. ASIC1a is supported by more direct evidence. Low-intensity ultrasound-induced neuronal activation was reduced by pharmacological inhibition or genetic suppression of ASIC1a in mouse brain, suggesting a causal contribution to *in vivo* ultrasound responsiveness (Lim et al., 2021). Because ASICs can interact with extracellular matrix and cytoskeletal components, their activation may involve tether-mediated mechanotransduction rather than direct sensing of lipid bilayer tension. Nevertheless, replication across brain regions, stimulation protocols, and species is needed before ASIC1a can be considered a general mediator of ultrasound neuromodulation.

#### 3.2.6 Convergence and evidence gaps

Mechanosensitive channels may contribute to both excitatory and inhibitory responses. Piezo1, TRPA1, TRPC6, TRPV4, and ASIC1a generally promote cation influx and depolarization, whereas TREK-1 and TRAAK favor K^+^ efflux and hyperpolarization. The net response is therefore likely determined by channel expression, cell type, membrane properties, acoustic parameters, and network context (Wan et al., 2024; Pirri, 2025). Current evidence does not support a single universal “ultrasound receptor.” Instead, multiple mechanosensitive pathways probably operate in parallel and converge on Ca^2+^ signaling, membrane excitability, and downstream transcriptional responses. Future studies should prioritize endogenous channel manipulation in intact brain tissue and distinguish native mechanotransduction from sonogenetic overexpression, general mechanical sensitivity, and theoretical predictions.

### 3.3 Calcium signaling

Intracellular calcium (Ca^2+^) signaling is a central link between ultrasound-induced mechanotransduction and downstream neural responses. As a ubiquitous second messenger, Ca^2+^ regulates membrane excitability, neurotransmitter release, synaptic plasticity, gene transcription, cellular metabolism, and survival (Bootman and Bultynck, 2020). In cortical neurons, focused ultrasound has been shown to induce mechanosensitive Ca^2+^ accumulation and ion-channel amplification, supporting a causal role for calcium signaling in ultrasound neuromodulation (Yoo et al., 2022). The resulting effects can be considered at three interconnected levels: initial Ca^2+^ mobilization, acute regulation of neuronal activity and neurotransmission, and longer-term changes in synaptic plasticity and gene expression.

#### 3.3.1 Ultrasound-induced calcium mobilization

A rapid increase in cytosolic Ca^2+^ is one of the most consistently observed cellular responses to low-intensity ultrasound. Calcium imaging studies in primary cortical cultures have demonstrated that ultrasound can evoke reproducible Ca^2+^ transients within seconds of stimulation (Bano et al., 2026). The initial response is primarily attributed to acoustic-force-induced deformation of the plasma membrane, which changes membrane tension and activates mechanically sensitive, calcium-permeable ion channels (Figure 5).

**Figure 5:**
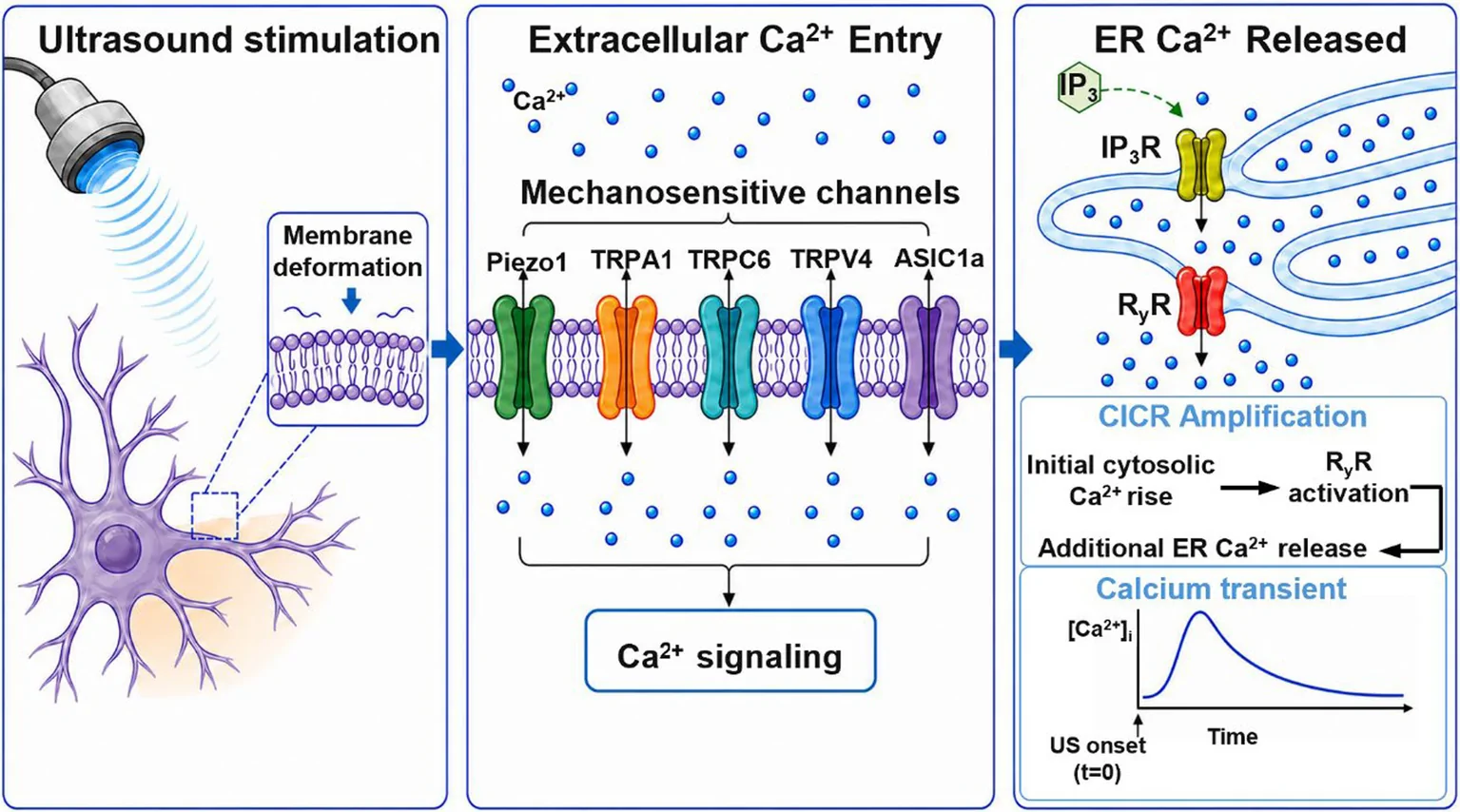
Ultrasound-induced calcium mobilization. Ultrasound-generated mechanical forces deform the plasma membrane and activate mechanosensitive ion channels, including Piezo1, TRPA1, TRPC6, TRPV4, and ASICs. The resulting increase in cytosolic Ca^2+^, together with Ca^2+^ release mediated by IP_3_ receptors, can activate R_y_R receptors and induce additional Ca^2+^ release from the endoplasmic reticulum through calcium-induced calcium release. The calcium-transient trace schematically represents the rapid increase and subsequent decline in normalized cytosolic Ca^2+^ after ultrasound onset and is not intended as a quantitative time course. ASIC, acid-sensing ion channel; Ca^2+^, calcium ion; [Ca^2+^]_i_, normalized cytosolic Ca^2+^; CICR, calcium-induced calcium release; IP_3_R, inositol 1,4,5-trisphosphate receptor; RyR, ryanodine receptor; TRPA1, transient receptor potential ankyrin 1; TRPV4, transient receptor potential vanilloid 4; TRPC6, transient receptor potential canonical 6.

Piezo-family channels provide a direct route for mechanically evoked cation entry and can couple membrane tension to intracellular signaling (Wu et al., 2017). TRP-family channels also contribute to ultrasound responsiveness in a cell-type-dependent manner. In particular, astrocytic TRPA1 activation has been implicated in ultrasound-induced Ca^2+^ signaling and subsequent modulation of neuronal activity (Oh et al., 2019). Thus, the magnitude and functional consequences of extracellular Ca^2+^ influx depend on the channel repertoire and physiological state of the stimulated cell.

Extracellular influx represents only one component of the calcium response. An initial increase in cytosolic Ca^2+^ can recruit intracellular stores by activating inositol 1,4,5-trisphosphate receptors (IP3Rs) and ryanodine receptors (RyRs) in the endoplasmic reticulum. Opening of these channels releases stored Ca^2+^ into the cytoplasm and may amplify the initial signal through calcium-induced calcium release (CICR) (Santulli et al., 2017). The combined contributions of extracellular entry and endoplasmic-reticulum release determine the amplitude, duration, and spatial distribution of the intracellular calcium transient. The amplitude and duration of the resulting Ca^2+^ transient vary with the acoustic exposure and cellular channel repertoire (Nandi et al., 2024). Consequently, ultrasound protocols that differ only in one or two parameters may generate distinct cellular responses. This parameter dependence should be considered when comparing experimental findings and when defining reproducible exposure conditions for neuromodulation.

#### 3.3.2 Acute calcium-dependent regulation of neuronal excitability and neurotransmission

Ultrasound-induced Ca^2+^ mobilization can rapidly alter neuronal excitability through both excitatory and inhibitory mechanisms. In hippocampal slice cultures and ex vivo mouse brain, low-intensity, low-frequency ultrasound activated voltage-gated sodium and calcium channels, evoked action potentials, and triggered synaptic transmission, providing direct evidence that ultrasound can recruit membrane conductances that support network excitation (Tyler et al., 2008). Nevertheless, the net electrophysiological response depends on the channel repertoire, membrane potential, cell type, and stimulation parameters. Therefore, the entry of Ca^2+^ should not be assumed to produce excitation under every condition.

Conversely, an increase in intracellular Ca^2+^ can activate calcium-dependent K^+^ channels. The resulting K^+^ efflux promotes membrane hyperpolarization and may reduce or terminate action-potential firing. Calcium-dependent changes in membrane potential therefore provide a mechanism through which the same initial calcium signal can produce excitation or inhibition (Elliott and Cooper, 2024). The direction of the response depends on the relative expression and activation of depolarizing conductances, calcium-activated K^+^ channels, and other ion channels in the stimulated population.

At presynaptic terminals, Ca^2+^ is also the immediate trigger for synaptic-vesicle exocytosis. Ultrasound-induced Ca^2+^ elevation can promote vesicle docking, membrane fusion, and neurotransmitter release (Bano et al., 2026). These processes may influence excitatory, inhibitory, and modulatory transmission, including signaling mediated by glutamate, γ-aminobutyric acid (GABA), dopamine, serotonin, and acetylcholine. The effects are therefore not limited to the firing of individual neurons but can extend to the neurochemical state of the targeted circuit. Modulation of dopaminergic signaling may be particularly relevant to basal-ganglia disorders, whereas changes in glutamatergic and GABAergic transmission may alter the excitation–inhibition balance in other neurological and psychiatric conditions (Singh and Reynolds, 2024).

Together, bidirectional regulation of firing and calcium-dependent transmitter release can rapidly modify oscillatory activity, neuronal synchronization, and functional connectivity. These immediate network consequences depend on which cellular elements are recruited and on whether excitatory, inhibitory, or neuromodulatory transmission predominates. Calcium signaling therefore provides a mechanistic basis for the context-dependent excitatory and inhibitory effects reported across ultrasound neuromodulation studies.

#### 3.3.3 Calcium-dependent synaptic plasticity and gene expression

Evidence linking ultrasound-evoked Ca^2+^ signals to synaptic plasticity and gene expression is accumulating, although the pathways involved depend on cell type and experimental conditions. In cultured hippocampal neurons, 15 min of 1-MHz low-intensity pulsed ultrasound (LIPUS) increased cytosolic Ca^2+^ by facilitating extracellular Ca^2+^ influx through L-type voltage-gated calcium channels. LIPUS also increased CaMKIIα phosphorylation and promoted the nuclear localization of phosphorylated CREB. Nifedipine prevented the CaMKIIα phosphorylation response, whereas either nifedipine or removal of extracellular Ca^2+^ prevented the nuclear accumulation of phosphorylated CREB, supporting LTCC-dependent CaMKII–CREB excitation-transcription coupling (Fan et al., 2024).

A distinct signaling profile has been observed in astrocytes. In cultured rat cortical astrocytes, LIPUS increased intracellular Ca^2+^, elevated CaMKII and CaMKIV protein levels, and enhanced TrkB and Akt phosphorylation. These changes were associated with increased BDNF mRNA and protein expression through Ca^2+^/CaMK- and TrkB/PI3K/Akt-dependent activation of NF-κB (Liu et al., 2017). The same study detected neither increased ERK1/2 phosphorylation nor CREB activation, indicating that ultrasound does not invariably recruit CaMK, ERK, Akt, and CREB as a single linear cascade.

ERK signaling has nevertheless been demonstrated under other stimulation conditions. Lim et al. showed that low-intensity ultrasound elicited Ca^2+^ responses in cultured cortical neurons through a mechanism involving acid-sensing ion channel 1a (ASIC1a) and the cytoskeleton. Transcranial stimulation also induced ERK phosphorylation in the mouse cortex, hippocampus, and amygdala, whereas ASIC1a deletion attenuated both the immediate ERK response and the increase in doublecortin-positive cells after repeated stimulation (Lim et al., 2021). In aged mice with postoperative neurocognitive impairment, hippocampal LIPUS increased ERK phosphorylation and restored BDNF, synaptophysin, and PSD95 expression together with improved spatial learning (Wang et al., 2023). Because intracellular Ca^2+^ was not directly quantified in the latter study, the proposed Piezo1/Ca^2+^/calpain-1/ERK sequence should be interpreted as an associated mechanism rather than a fully established causal pathway.

Functional evidence for ultrasound-induced long-term plasticity is also becoming more direct. In mouse hippocampal slices, selective stimulation of CA3 produced sustained potentiation of CA3-CA1 field excitatory postsynaptic potentials, and the probability and magnitude of this acoustic long-term potentiation increased with acoustic pressure (Zhou et al., 2025). In adult rats, repeated low-intensity focused ultrasound targeting the dentate gyrus increased BDNF, FGF-2, and Sox2 mRNA expression, enhanced neuronal proliferation and differentiation, and improved recognition memory in a frequency-dependent manner, with the strongest effects at 0.5 MHz (Kanaan et al., 2026). These findings establish ultrasound-responsive synaptic potentiation and plasticity-related transcription, but they do not yet demonstrate that every long-term effect is mediated by one Ca^2+^-dependent molecular pathway.

Overall, calcium-dependent signaling appears to contribute to ultrasound-induced synaptic and transcriptional plasticity, but the available evidence does not support a single universal signaling cascade. Receptor trafficking, dendritic-spine remodeling, and long-term depression have not yet been directly demonstrated and should therefore be considered potential rather than established effects of ultrasound stimulation.

### 3.4 Glial cell responses

Ultrasound neuromodulation is often interpreted primarily in terms of direct neuronal activation or inhibition, but changes in neural-circuit activity may also involve responses from glial cells. Astrocytes regulate extracellular ion and neurotransmitter homeostasis, synaptic transmission, and neurovascular coupling; microglia coordinate immune surveillance, inflammatory signaling, and activity-dependent synaptic remodeling; and oligodendroglial-lineage cells support axonal conduction and adaptive myelination (Kedzia et al., 2026; Micali et al., 2026; Huang M. et al., 2025). These functions place glial cells at the interface between acoustic mechanotransduction and both the immediate and delayed effects of ultrasound on neural networks.

#### 3.4.1 Astrocytes: active participants in ultrasound neuromodulation

Astrocytes are the most abundant glial cell type in the central nervous system and are increasingly recognized as active regulators of neural information processing. Through extensive interactions with neurons, synapses, and blood vessels, astrocytes participate in the formation of the so-called tripartite synapse, where they influence neurotransmission and synaptic plasticity. Astrocytes are highly responsive to mechanical stimulation. Similar to neurons, astrocytes express multiple mechanosensitive ion channels, including Piezo1, TRPA1, TRPV4, and various stretch-activated channels.

Astrocytes exhibit direct ultrasound-evoked Ca^2+^ responses, but the underlying channel contribution is parameter- and cell-type-dependent. In cultured astrocytes, 250-kHz focused ultrasound evoked Ca^2+^ signals, and pharmacological blockade of mechanosensitive and transient receptor potential-related channels reduced response prevalence or amplitude (Newman et al., 2024). However, direct evidence that localized ultrasound initiates long-range intercellular astrocytic Ca^2+^ waves *in vivo* remains limited, and widespread functional-connectivity changes should not be attributed to this mechanism without concurrent cellular measurements.

Astrocytes can also influence neuronal excitability through ultrasound-dependent regulation of extracellular neurotransmitters. In cultured neural networks, repetitive pulsed-wave ultrasound suppressed recurrent bursting by increasing ambient GABA and tonic extrasynaptic GABA-A receptor currents; this effect was absent in astrocyte-free cultures and was prevented by TRPA1 blockade (Mishima et al., 2024). This study provides direct evidence for an astrocyte-dependent inhibitory pathway. By contrast, ultrasound-specific evidence for astrocytic release of glutamate, ATP, or D-serine remains less complete and should not be inferred solely from their established roles in gliotransmission.

#### 3.4.2 Microglia: ultrasound and neuroimmune regulation

Ultrasound can modify microglial behavior, but the direction and magnitude of the response depend on acoustic parameters and disease context (Grewal et al., 2026; Grewal et al., 2023). Microbubble-assisted BBB opening can also alter microglial activity and pathology (Shen et al., 2020). This intervention has a distinct mechanism, it should not be used as direct evidence for non-ablative neuromodulation. The NLRP3 inflammasome is an important mediator of neuroinflammation (Chen et al., 2023), but direct evidence that non-ablative ultrasound neuromodulation suppresses brain NLRP3 activity through Ca^2+^ signaling remains limited. Accordingly, NLRP3 modulation is presented here as a hypothesis requiring pathway-specific inhibition or genetic validation rather than as an established mechanism.

#### 3.4.3 Oligodendrocytes and myelin remodeling

Evidence that ultrasound affects oligodendrocytes and myelin is emerging from disease models. In cuprizone-treated mice, LIPUS increased cortical and corpus-callosum levels of myelin basic protein and neural/glial antigen 2 relative to sham treatment (Guo et al., 2021). In a mouse model of focal cerebral ischemia, repeated LIPUS protected oligodendrocyte precursor cells during the acute phase and subsequently increased newly generated mature oligodendrocytes and myelin proteins, with changes associated with down-regulation of IL-17A/Notch1 signaling (Wang et al., 2025). These studies support an oligodendroglial contribution to ultrasound-associated white-matter repair, although cell-autonomous mechanisms still require lineage-specific manipulation and longitudinal myelin assessment.

## 4 Therapeutic applications in brain disorders

The unique ability of ultrasound neuromodulation to non-invasively target deep brain structures with high spatial precision has generated considerable interest in its therapeutic application across a wide spectrum of neurological and psychiatric disorders. Beyond transient modulation of neuronal activity, ultrasound can influence neuroplasticity, neuroinflammation, neurovascular function, and protein clearance pathways, thereby addressing multiple pathogenic mechanisms simultaneously (Figure 6). Recent preclinical and clinical studies suggest that ultrasound-based neuromodulation may provide disease-modifying effects in several brain disorders (Mitterwallner et al., 2026). Although the underlying mechanisms vary among diseases, many therapeutic outcomes appear to converge on common biological pathways involving calcium signaling, glial regulation, neurovascular coupling, and neural circuit remodeling.

**Figure 6:**
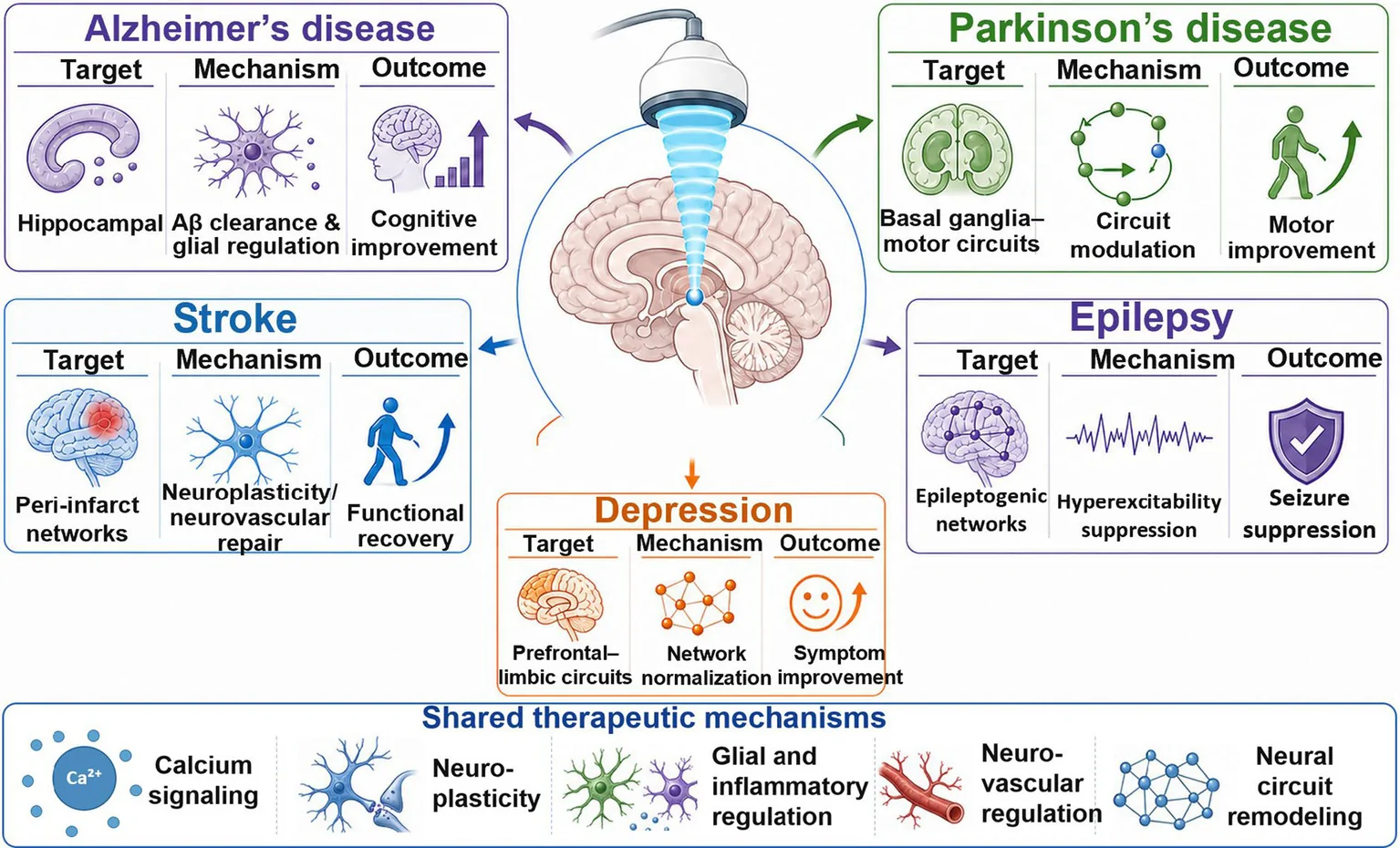
Therapeutic applications of ultrasound-based interventions in brain disorders. Schematic summary of the potential applications of ultrasound neuromodulation in Alzheimer’s disease, Parkinson’s disease, stroke, epilepsy, and depression. Ultrasound can target disease-relevant regions and circuits, including hippocampal networks in Alzheimer’s disease, basal ganglia–thalamocortical and motor circuits in Parkinson’s disease, peri-infarct networks after stroke, epileptogenic circuits in epilepsy, and prefrontal–limbic networks in depression. The proposed therapeutic effects include modulation of neuronal activity and synaptic plasticity, regulation of glial and inflammatory responses, promotion of vascular and neural repair, suppression of network hyperexcitability, and normalization of dysfunctional mood-related circuits. These mechanisms contribute to cognitive improvement, motor regulation, functional recovery, seizure suppression, and improvement of depressive symptoms.

### 4.1 Alzheimer’s disease

Alzheimer’s disease (AD) is the most prevalent neurodegenerative disorder and is characterized by progressive cognitive decline, extracellular amyloid-β (Aβ) deposition, intracellular tau pathology, synaptic dysfunction, neuroinflammation, and cerebral hypoperfusion (Angelopoulou et al., 2024). Despite decades of research, currently available pharmacological therapies provide only modest symptomatic benefits and do not fully halt disease progression.

#### 4.1.1 Hippocampal activation and cognitive enhancement

The hippocampus and distributed memory networks are affected early in AD. Preclinical studies indicate that ultrasound can modulate hippocampal excitability and plasticity-related signaling (Niu et al., 2022). Clinical transcranial pulse stimulation (TPS), however, has generally targeted distributed cortical AD networks rather than the hippocampus alone. In an uncontrolled follow-up study of 17 patients, improvement in neuropsychological scores correlated with cortical-thickness changes in AD-relevant regions after TPS (Popescu et al., 2021). A subsequent randomized, double-blind, sham-controlled crossover trial found no significant verum-versus-sham difference in the primary cognitive outcome for the full cohort, although exploratory analyses suggested benefit in younger participants and showed changes in functional activation and connectivity (Matt et al., 2025). Thus, clinical target engagement is encouraging, but efficacy and optimal network targeting require further confirmatory trials.

#### 4.1.2 Amyloid-β clearance

One of the most extensively investigated mechanisms of ultrasound therapy in AD involves enhancement of Aβ clearance (Ma et al., 2023). Preclinical studies have demonstrated that low-intensity ultrasound stimulation can significantly reduce amyloid plaque burden in transgenic AD mouse models (Su et al., 2025). Multiple mechanisms appear to contribute to this effect. First, ultrasound promotes microglial activation and phagocytic clearance of Aβ aggregates. Enhanced microglial recruitment around plaques facilitates removal of extracellular amyloid deposits (Grewal et al., 2023). Second, ultrasound-induced alterations in interstitial fluid dynamics may improve glymphatic clearance pathways, thereby facilitating elimination of soluble Aβ species from the brain (Xiao et al., 2025). Third, transient modulation of blood–brain barrier permeability observed under specific ultrasound conditions may enhance clearance of pathological proteins and improve delivery of therapeutic agents (Gorick et al., 2022).

#### 4.1.3 Reduction of neuroinflammation

Chronic neuroinflammation contributes to AD progression (Singh, 2022). Direct evidence for an anti-inflammatory effect of non-ablative ultrasound has recently been reported in 5xFAD AD model mice. In awake and freely moving female 5xFAD mice, a 14-day regimen of transcranial ultrasound stimulation pulsed at 40 Hz reduced IL-6, IL-1β, and TNF-α, down-regulated RIPK1 and phosphorylated NF-κB, increased plaque-associated microglial activity, reduced Aβ deposition, and improved performance in the Y-maze and Morris water maze (Yi et al., 2026). These findings suggest that the cognitive benefits observed under the tested exposure conditions can be associated with modulation of neuroinflammatory and necroptotic pathways, possibly involving RIPK1/NF-κB signaling. Studies using microbubbles to open the BBB involve a distinct intervention and should be evaluated separately (Shen et al., 2020). Direct suppression of NLRP3 by non-ablative ultrasound in AD remains to be established.

### 4.2 Parkinson’s disease

Parkinson’s disease (PD) is characterized by degeneration of dopaminergic neurons within the substantia nigra pars compacta and dysfunction of basal ganglia-thalamocortical circuits (McGregor and Nelson, 2019). The resulting abnormalities manifest clinically as bradykinesia, rigidity, resting tremor, and postural instability.

The primary motor cortex is an important output node of the basal ganglia–thalamocortical network, and its excitability is altered in PD (McGregor and Nelson, 2019). In a sham-controlled crossover study of 24 participants, Zhang et al. applied 500-kHz repetitive transcranial ultrasound stimulation to the left primary motor cortex and observed increased motor-evoked potential amplitudes that persisted for at least 30 min (Zhang Y. et al., 2021). These findings demonstrate that ultrasound can induce sustained changes in human motor-cortex excitability and may therefore engage a circuit node relevant to PD. However, because the participants were neurologically healthy, whether this physiological modulation improves bradykinesia, rigidity, gait, or other motor symptoms in PD requires direct evaluation in patient cohorts.

A major advantage of ultrasound neuromodulation is its potential to reach deep brain structures non-invasively, including components of basal-ganglia circuits. Evidence for disease modification remains preclinical. In a 6-hydroxydopamine rat model of PD, repeated LIPUS improved locomotor performance, preserved tyrosine-hydroxylase-positive nigrostriatal neurons, increased glial cell line-derived neurotrophic factor in the substantia nigra pars compacta, and reduced lipocalin-2 and IL-1β (Sung et al., 2021). These results support neurotrophic and anti-inflammatory effects in this model but do not establish equivalence to deep brain stimulation or clinical disease-modifying efficacy.

### 4.3 Stroke

Stroke remains a leading cause of long-term disability worldwide. Functional deficits following stroke arise from neuronal death, neuroinflammation, white matter injury, vascular dysfunction, and disruption of neural network connectivity (Irisa and Shichita, 2025). Among neurological disorders, stroke may represent one of the most promising applications of ultrasound neuromodulation because recovery depends heavily on neuroplasticity and circuit reorganization.

Experimental evidence indicates that ultrasound can support recovery after ischemic injury through vascular and neurogenic mechanisms. In mice subjected to middle cerebral artery occlusion, whole-brain LIPUS increased VEGF and phosphorylated endothelial nitric oxide synthase, enhanced peri-infarct angiogenesis and striatal neurogenesis, and improved tightrope and rotarod performance; these benefits were absent in endothelial nitric oxide synthase-deficient mice (Ichijo et al., 2021). This study provides direct pathway-level evidence for endothelial nitric oxide synthase-dependent angio-neurogenesis, whereas the relative contribution of neuronal Ca^2+^ signaling and dendritic remodeling remains to be determined.

Additional stroke studies support parameter and timing dependent neuroprotection and repair. In a mouse middle cerebral artery occlusion model, low-intensity ultrasound reduced infarct size and increased phosphorylated endothelial nitric oxide synthase with only marginal effects on cerebral blood flow (Davis et al., 2023). In a separate mouse study initiated during the late acute stage, low-intensity focused ultrasound improved cerebral blood flow and neurobehavioral outcomes and promoted angiogenesis and synaptogenesis through an astrocyte-associated HMGB1/CAMK2N1 response (Qi et al., 2024). These findings support a therapeutic window beyond immediate injury, but translation will require standardized exposure estimates, clinically relevant timing, and replication in larger models.

### 4.4 Epilepsy

Epilepsy is characterized by recurrent seizures arising from abnormal hypersynchronous neural activity (Huang N. et al., 2025). Low-intensity ultrasound has suppressed epileptiform activity in acute and chronic experimental models (Zou et al., 2020; Afif et al., 2023; Lin et al., 2020). In a kainic-acid rat model, a single session of pulsed focused ultrasound reduced electroencephalographic spikes, bursts, and electrographic seizures for up to seven weeks, although the most intense regimen produced inflammation and gliosis (Chu P. C. et al., 2022). These data support durable, dose-dependent network inhibition while also emphasizing the need to define a safety–efficacy window.

### 4.5 Depression and other psychiatric disorders

Major depressive disorder (MDD) is increasingly conceptualized as a network disorder involving imbalanced frontoparietal and limbic activity (Lee, 2025). Yaakub et al. show that transcranial focused ultrasound can alter neurochemistry and functional connectivity in deep cortical regions (Yaakub et al., 2023), supporting circuit-level target engagement. In rats exposed to prolonged restraint stress, two weeks of low-intensity transcranial ultrasound targeting the prelimbic cortex reversed anhedonia and increased hippocampal BDNF without histological injury (Zhang et al., 2019). These findings provide preclinical evidence for antidepressant-like effects but do not establish efficacy across mood, anxiety, obsessive-compulsive, or trauma-related disorders.

## 5 Clinical translation and safety

### 5.1 Clinical trials

The clinical translation of ultrasound-based brain interventions has advanced along three mechanistically distinct pathways: non-ablative neuromodulation, thermal ablation, and microbubble-assisted BBB opening (Table 1). Encouraging findings from animal models have stimulated growing interest in evaluating the therapeutic potential of low-intensity tFUS and TPS in patients with neurological and psychiatric disorders (Attali et al., 2025; Kim et al., 2024b; Hu Y. Y. et al., 2023). Although most clinical studies remain exploratory in nature, accumulating evidence suggests that ultrasound neuromodulation can safely influence neural activity, modulate dysfunctional brain circuits, and potentially improve clinical outcomes across multiple disease settings.

**Table 1:** Representative clinical studies of mechanistically distinct ultrasound-based interventions in brain disorders.

Intervention class	Disease	Ultrasound approach	Target	Clinical status	Findings	Safety and limitations	NCT No.
Non-ablative ultrasound neuromodulation	Alzheimer’s disease	TPS, tFUS	hippocampal and default mode network circuits	Exploratory clinical studies and emerging randomized trials	No significant verum-versus-sham difference in the full-cohort primary cognitive outcome, with exploratory benefit in participants aged ≤70 years and secondary functional-imaging change	mild transient adverse events;	03770182, 05910619, 07143734
	Parkinson’s disease	MR-guided FUS, low-intensity tFUS	Basal ganglia-thalamocortical motor circuits; motor cortex	Small clinical studies; ongoing trials	Preliminary studies suggest effects on motor performance, tremor severity, and motor cortical excitability	Small cohorts; optimal targets, parameters, and durability remain uncertain	04593875, 06090292, 06259708
	Major depressive disorder	Low-intensity tFUS	Dorsolateral prefrontal cortex; anterior cingulate cortex;	Pilot and sham-controlled clinical studies; ongoing trials	Reported improvements in depressive symptoms and modulation of functional connectivity in preliminary studies	Requires larger randomized trials and standardized stimulation protocols	04405791, 06013384, 06085950, 06320028
	Epilepsy	Low-intensity tFUS	Epileptogenic cortical networks and seizure-related circuits	Pilot safety and feasibility studies; ongoing clinical development	Early studies suggest modulation of cortical excitability and large-scale network activity	Long-term antiseizure efficacy	02151175, 03868293, 05947656, 06388707
Ultrasound thermal ablation	Tremor-dominant Parkinson’s disease	MR-guided FUS thermal ablation	Ventral intermediate nucleus of the thalamus	Clinical trial / clinical adoption for tremor-dominant cases	Reduced medication-refractory tremor in selected patients	Ablative lesioning	01772693
	Parkinson’s disease	MR-guided FUS subthalamotomy	Subthalamic nucleus	Randomized sham-controlled clinical trial	Improved motor signs on the treated side in selected patients	dyskinesia, gait/speech effects, and lesion-related deficits	03454425
	Parkinson’s disease with dyskinesia	MR-guided FUS pallidotomy	Globus pallidus internus	Randomized clinical trial	Improved motor signs or dyskinesia-related outcomes in selected patients	Ablation-related adverse effects and durability require ongoing evaluation	03319485
Microbubble-assisted focused ultrasound BBB opening	Alzheimer’s disease	microbubble-assisted FUS-mediated BBB opening	Targeted cortical / hippocampal / memory-related regions	Early-phase clinical studies	feasible and reversible BBB opening; explored potential enhancement of amyloid clearance	Edema, microhemorrhage, cavitation-related effects	02986932, 03119961, 03671889, 04118764

Among neurodegenerative disorders, AD is one of the most extensively investigated indications. Early uncontrolled TPS studies reported cognitive and functional-connectivity changes (Chen et al., 2024; Beisteiner et al., 2020), whereas a subsequent randomized crossover trial did not show a significant verum-versus-sham difference in the primary cognitive outcome for the full cohort, despite exploratory benefit in younger participants (Matt et al., 2025). In PD, preliminary studies have examined cortical and subcortical motor targets (Kofoed et al., 2026; Lin et al., 2026). Theta-burst transcranial ultrasound stimulation of the motor cortex increased motor-evoked-potential amplitudes in healthy controls and in patients with PD while on, but not off, dopaminergic medication; an exploratory bradykinesia improvement was likewise observed only in the on-medication state (Grippe et al., 2024). These findings demonstrate state-dependent target engagement and plasticity, but they do not yet establish ultrasound as a replacement for deep brain stimulation.

Clinical investigation of ultrasound neuromodulation in psychiatric disorders is expanding. Human studies have shown modulation of prefrontal, cingulate, amygdala, and related networks (di Biase et al., 2019; Chou et al., 2024; Sanguinetti et al., 2020). In a randomized, double-blind, sham-controlled trial in patients with MDD, six sessions of low-intensity transcranial focused ultrasound targeting the left dorsolateral prefrontal cortex were well tolerated and were associated with improved Montgomery-Asberg Depression Rating Scale scores and altered subgenual anterior cingulate connectivity (Oh et al., 2024). A pilot randomized trial of TPS in adults with depressive symptoms also reported reduced symptom scores, although its small sample and exploratory design limit efficacy conclusions (Cheung et al., 2023; Matt et al., 2024; Sarica et al., 2022). Overall, current data support feasibility and short-term target engagement, while disorder-specific efficacy and durability require larger preregistered trials.

### 5.2 Safety considerations

Safety remains a critical prerequisite for the widespread clinical adoption of ultrasound neuromodulation. Compared with invasive neuromodulation techniques, ultrasound offers the significant advantage of non-invasive energy delivery without surgical implantation or direct tissue penetration. Nevertheless, the interaction between acoustic energy and biological tissue raises important safety considerations that require careful evaluation.

Thermal safety must be evaluated from the complete acoustic exposure rather than from a nominal label of low intensity. A systematic review found adverse tissue effects to be rare and largely associated with high stimulation intensity or rate, but it also emphasized heterogeneous reporting and incomplete safety margins (Pasquinelli et al., 2019). Retrospective simulations across rodent protocols estimated negligible-to-modest heating for most settings but identified one protocol with a potentially consequential temperature rise (Constans et al., 2018). In living macaques, a transcranial MR acoustic-radiation-force imaging protocol was implemented with less than 0.1 °C focal heating under the tested conditions (Phipps et al., 2019). Conversely, focused ultrasound-induced suppression of somatosensory evoked potentials in another experimental preparation tracked an approximately 2 °C temperature increase, demonstrating that thermal neuromodulation can occur below ablative temperatures (Darrow et al., 2019). Accordingly, exposure-specific acoustic and thermal modeling, measurement where feasible, and transparent reporting are required for each protocol.

Cavitation represents another important safety consideration. Cavitation refers to the oscillation or collapse of gas-filled bubbles in response to acoustic pressure fluctuations. While inertial cavitation can generate substantial mechanical stress and tissue damage, neuromodulation protocols are typically designed to operate below cavitation thresholds (Radjenovic et al., 2022; Tao et al., 2026). Passive cavitation monitoring and acoustic safety assessments have demonstrated that appropriately selected stimulation parameters can minimize cavitation-related risks (Tao et al., 2026; Shinar and Ilovitsh, 2025). Nevertheless, continued monitoring remains important, particularly when combining ultrasound with microbubbles or blood–brain barrier opening strategies.

Potential tissue injury and adverse symptoms of ultrasound intervention have been investigated in animals and humans. In a retrospective analysis of 120 participants across seven human low-intensity focused ultrasound studies, 64 completed follow-up safety questionnaires. No serious adverse events were identified, and 7 respondents reported mild-to-moderate symptoms considered possibly or probably related to participation, with no new symptoms at follow-up to one month (Legon et al., 2020). This evidence supports short-term tolerability under the evaluated protocols but does not establish long-term safety for repeated therapeutic exposure.

In addition, the International Transcranial Ultrasonic Stimulation Safety and Standards (ITRUSST) consortium has recently proposed a consensus framework for evaluating the biophysical safety of transcranial ultrasound stimulation. For individuals without specific vascular or thermoregulatory vulnerabilities and without administration of ultrasound contrast agents, Aubry et al. defined conditions associated with nonsignificant biophysical risk using mechanical and thermal exposure metrics, including the mechanical index, transcranial mechanical index, temperature elevation, thermal dose, and thermal index (Aubry et al., 2025). These consensus levels should guide protocol-specific risk assessment but should not be interpreted as definitive safety limits or substitutes for ethical and regulatory review.

## 6 Discussion: challenges and future perspectives

### 6.1 Mechanistic challenges

Despite rapid advances in the field, the fundamental mechanisms underlying ultrasound neuromodulation remain incompletely understood. One of the most significant unresolved questions concerns the identity of the primary molecular mediators responsible for converting acoustic energy into biological responses. Although mechanosensitive ion channels such as Piezo1, TRPA1, TRPV4, and TREK-family potassium channels have emerged as leading candidates (Chu Y. C. et al., 2022; Zhu et al., 2023), their relative contributions under different stimulation conditions remain unclear. Existing evidence suggests that multiple mechanotransduction pathways may operate simultaneously, with channel recruitment varying according to cell type, brain region, and acoustic parameters (Cheng et al., 2026; Magaram et al., 2022).

Additional uncertainty surrounds the physical mechanisms responsible for initiating neuromodulatory effects. Acoustic radiation force has received substantial experimental support and is widely regarded as one of the leading candidate mechanisms for low-intensity ultrasound neuromodulation. In contrast, the intramembrane cavitation hypothesis provides an alternative theoretical framework capable of explaining neuronal activation under low acoustic intensities. However, direct experimental evidence confirming intramembrane cavitation in living neural tissue remains limited. Future studies integrating electrophysiology, advanced imaging, molecular biology, and computational modeling will be required to establish a unified mechanistic framework.

### 6.2 Technical challenges

Several technical barriers limit the precision and reproducibility of ultrasound neuromodulation. The human skull causes attenuation, reflection, refraction, phase distortion, and target-dependent standing-wave patterns. CT- and MR-derived simulations across 12 human skulls demonstrated substantial interindividual variation in intracranial pressure fields and showed that no simple linear relationship adequately characterized pressure maps across participants (Mueller et al., 2017). Although CT-based phase correction and phased-array technologies can improve transcranial focusing (Yin et al., 2023), individualized exposure estimation remains important. Neuromodulatory outcomes are also sensitive to frequency, pressure, pulse duration, duty cycle, sonication duration, target, and brain state.

### 6.3 Clinical and regulatory challenges

Despite a growing number of clinical investigations, ultrasound neuromodulation remains at an early stage of translation. Most published studies are pilot trials, feasibility studies, or small randomized investigations with limited statistical power (Darmani et al., 2022; Davidson et al., 2024; Matt et al., 2024). Short follow-up periods further restrict conclusions regarding the durability of therapeutic effects and the safety of repeated or long-term exposure. Substantial heterogeneity also exists across stimulation protocols, including differences in acoustic frequency, pressure, pulse duration, duty cycle, sonication schedule, target selection, and skull-correction procedures. This variability complicates comparisons across studies and prevents reliable characterization of dose–response relationships. In addition, outcome measures vary considerably among trials, and few studies combine standardized clinical endpoints with objective neurophysiological or imaging biomarkers.

Future clinical studies should therefore adopt harmonized reporting standards for acoustic exposure, estimated *in situ* dose, targeting accuracy, treatment schedule, and adverse events. Multicenter, sham-controlled trials with preregistered endpoints and extended follow-up will be required to establish efficacy and long-term safety. Regulatory translation presents additional challenges. Regulatory pathways for ultrasound neuromodulation devices are still evolving (Almarie and Fregni, 2025). Although focused ultrasound systems have been approved for selected ablative indications, the corresponding regulatory precedents cannot be directly applied to non-ablative neuromodulation. Regulatory assessment is further complicated by the multidimensional nature of acoustic dosing and the lack of standardized exposure thresholds. Existing ITRUSST recommendations provide an initial framework for improving methodological consistency in transcranial ultrasound research. Martin et al. proposed a minimum reporting checklist covering the transducer and drive system, device settings, free-field acoustic parameters, pulse-timing parameters, estimated *in situ* brain exposure, and intensity metrics (Martin et al., 2024). The IFCN-endorsed practical guide developed by Murphy et al. further addressed transducer selection, experimental design, control conditions, treatment planning, and the integration of ultrasound with neuroimaging and electrophysiology (Murphy et al., 2025). Consistent adoption and continued refinement of these recommendations, together with standardized clinical outcomes and long-term safety monitoring, should improve reproducibility and support regulatory evaluation.

### 6.4 Future perspectives

Closed-loop systems integrating electrophysiological or neuroimaging feedback enable adaptive ultrasound delivery (Karthick Raghunath et al., 2025). Artificial intelligence may also accelerate patient-specific treatment planning, but this application requires ultrasound-specific validation rather than extrapolation from unrelated imaging biomarkers (Kim, 2021). TUSNet, an end-to-end deep-learning model trained on synthetic skull CT segments and tested on real skull CT segments, predicted two-dimensional pressure fields and phase-aberration corrections in 21 ms, with a mean focal-positioning error of 0.18 mm relative to k-Wave simulations (Naftchi-Ardebili et al., 2026). Because validation used simulated, noise-free acoustic fields, experimental and prospective clinical validation remains necessary before deployment. Sonogenetics may further increase cellular specificity by combining ultrasound with engineered mechanosensitive proteins. BBB opening and ultrasound-assisted drug delivery are also promising but constitute mechanistically distinct interventions and should be evaluated separately from non-ablative neuromodulation.

## 7 Summary

Ultrasound neuromodulation combines non-invasive delivery, millimeter-scale targeting, deep-brain accessibility, reversibility, and flexible acoustic control. Preclinical evidence indicates that its effects arise from coordinated mechanical, ion channel, calcium-dependent, glial, and neurovascular mechanisms, whereas early human studies support its feasibility and short-term safety across several neurological and psychiatric conditions. However, clinical efficacy remains uncertain because most trials involve small cohorts, heterogeneous stimulation protocols, short follow-up, and non-standardized outcomes. Key priorities include defining *in situ* acoustic dose, improving skull-specific targeting, establishing disease-specific parameter windows, and evaluating long-term safety in adequately powered multicenter trials. Integration with closed-loop monitoring, individualized computational modeling, and cell-specific strategies may facilitate the development of ultrasound neuromodulation from an experimental technique into a reproducible platform for precision neurotherapeutics.
